# Sequence Variations Within *HLA-G* and *HLA-F* Genomic Segments at the Human Leukocyte Antigen Telomeric End Associated With Acute Graft-Versus-Host Disease in Unrelated Bone Marrow Transplantation

**DOI:** 10.3389/fimmu.2022.938206

**Published:** 2022-07-21

**Authors:** Shingo Suzuki, Satoko Morishima, Makoto Murata, Masafumi Tanaka, Atsuko Shigenari, Sayaka Ito, Uma Kanga, Jerzy K. Kulski, Yasuo Morishima, Takashi Shiina

**Affiliations:** ^1^ Department of Molecular Life Science, Tokai University School of Medicine, Isehara, Japan; ^2^ Division of Endocrinology, Diabetes and Metabolism, Hematology, Rheumatology, Second Department of Internal Medicine, Graduate School of Medicine, University of the Ryukyus, Nishihara, Japan; ^3^ Department of Hematology and Oncology, Nagoya University Graduate School of Medicine, Nagoya, Japan; ^4^ Clinical Immunogenetics Laboratory, Centre for Excellence in Molecular Medicine, Department of Transplant Immunology and Immunogenetics, All India Institute of Medical Sciences, New Delhi, India; ^5^ Faculty of Health and Medical Sciences, The University of Western Australia Medical School, Crawley, WA, Australia; ^6^ Department of Promotion for Blood and Marrow Transplantation, Aichi Medical University School of Medicine, Nagakute, Japan; ^7^ Department of Hematology and Oncology, Nakagami Hospital, Okinawa, Japan

**Keywords:** human leukocyte antigen, graft-versus-host disease, bone marrow transplantation, haplotype, hitchhiking diversity, genotyping

## Abstract

Acute graft-versus-host disease (aGVHD) is defined as a syndrome of an immunological response of graft to the host that occurs early after allogeneic hematopoietic stem cell transplantation (HCT). This disease is frequently observed even in HCT matched for human leukocyte antigen (HLA) alleles at multiple gene loci. Although the HLA region represents complex and diverse genomic characteristics, detailed association analysis is required for the identification of uncharacterized variants that are strongly associated with aGVHD. We genotyped three loci, *OR2H2*, *HLA-F-AS1*, and *HLA-G*, that are located in the 460 kb of HLA telomeric region and statistically analyzed the genotypes including *HLA-DPB1* with clinical and transplantation outcomes using 338 unrelated bone marrow transplantation (UR-BMT) patient–donor pairs who were matched for *HLA-A*, *HLA-B*, *HLA-C*, *HLA-DRB1*, and *HLA-DQB1* (HLA-10/10). Multivariate analyses demonstrated that *HLA-F-AS1* and *HLA-DPB1* mismatches were associated with grade II–IV aGVHD (hazard ratio (HR), 1.76; 95% CI, 1.07–2.88; p = 0.026; and HR, 1.59; CI, 1.02–2.49; p = 0.042, respectively). There was no confounding between *HLA-F-AS1* and *HLA-DPB1* (p = 0.512), suggesting that the *HLA-F-AS1* mismatch has a strong effect on aGVHD independently of *HLA-DPB1*. Moreover, a stratified analysis suggested possible associations of *HLA-F-AS1*, *HLA-DPB1*, and/or *HLA-G* mismatches with grade II–IV aGVHD and the more severe grade III–IV aGVHD. These findings provide new insights into understanding the molecular mechanism of aGVHD caused by HLA-matched UR-BMT.

## Introduction

Unrelated donor hematopoietic stem cell transplantation (UR-HCT) is an established curative therapy for patients with hematologic malignancies and other hematologic and immunologic disorders in cases where no human leukocyte antigen (HLA)-matched relatives are found. An HLA mismatch between a donor’s cells or tissues (graft) transplanted into an unrelated patient (host) is well known to cause acute graft-versus-host disease (aGVHD) in many cases of UR-HCT (1, 2). The onset of aGVHD usually is defined as a syndrome characterized by rash, jaundice, and diarrhea, which occur early after allogeneic HCT with an immunological response of the graft to the host, in a graft-versus-host (GVH) direction, which may lead to graft rejection and death of the transplant recipient (3). The severity of aGVHD is graded between I and IV, with grade I the least severe (clinical manifestations limited to less than 50% of body skin involvement) and grade IV the most severe (skin, gastrointestinal tract, and/or liver involvement) stage prior to mortality (4).

Numerous association analyses between the HLA genotypes within the major histocompatibility complex (MHC) genomic region on chromosome 6 (6p21.3) and clinical and transplantation outcomes have reported that aGVHD strongly associates with patient–donor HLA mismatches (5–11). Therefore, the primary strategy to avoid aGVHD development is to match the HLA gene loci and alleles between donors and recipients (HLA-matched transplantation), so that, for example, *HLA-A*, *HLA-B*, *HLA-C*, and *HLA-DRB1* (HLA-8/8) or *HLA-A*, *HLA-B*, *HLA-C*, *HLA-DRB1*, and *HLA-DQB1* (HLA-10/10) are matched between donors and recipients in a clinical setting (12–15). However, 11% of recipients with HLA-8/8-matched UR-HCT and 17% of recipients with HLA-10/10-matched-related HCT or UR-HCT develop grade III–IV aGVHD (2, 16). This means that there are uncharacterized variations involved in the development of aGVHD in addition to the classical HLA loci traditionally used for HLA-matched HCT. Since the MHC genomic region is composed of more than 400 genes together with the classical HLA class I and class II genes, it is possible that one or more of the unidentified neighboring genes were unmatched and might have contributed to aGVHD and the rejection of the graft.

In the HLA genomic region, *HLA-DPB1* has been characterized as an aGVHD-related HLA locus, as follows: 1) *HLA-DPB1* mismatch associates with severe aGVHD (15, 17), 2) T-cell epitope (TCE) algorism of DPB1 (18), 3) highly expressed *HLA-DPB1* alleles associate with aGVHD (19), and 4) evolutionarily highly conserved gene structures in *HLA-DPB1* associate with aGVHD, which are different from the TCE algorism (20). Hence, the importance of *HLA-DPB1* for transplantation medicine is increasingly recognized with suggestions that *HLA-DPB1* matched donors should be added to the traditional HLA-10/10 genotyping set. Also, the non-classical MHC gene, *HLA-G*, is known to associate with transplantation outcomes; for example, the insertion alleles of 14-bp insertion or deletion (indel) polymorphism (rs371194629) in the 3′-untranslated region (3′UTR) of *HLA-G* were reported to associate with aGVHD (21).

*HLA-G* and *HLA-F* genes are both located on the telomeric end of the MHC genomic region that represents a haplotypic segment with diverse genomic characteristics and that might be associated with aGVHD but has yet not been analyzed in detail (22). The HLA-G molecule binds to inhibitory receptors such as leukocyte Ig-like receptors B1 and B2 (LILRB1 and LILRB2), and the molecule has immunological functions such as inhibition of the cytotoxic activity of natural killer (NK) cells, induction of apoptosis and dendritic cells, and inhibition of CD4^+^ T-cell proliferation and antigen-presenting cells (23–27). Although the biological function of *HLA-F* has not been investigated in the same detail or as broadly as *HLA-G*, *HLA-F* alleles and *HLA-F* gene expression were identified to be involved with pathological and physiological processes such as viral infection, glioblastoma, alloimmunization in pregnancy, and autoimmune diseases like systemic lupus erythematosus (SLE) and breast cancer (28–32). The HLA-F molecule binds to inhibitory killer immunoglobulin-like receptors (KIRs) (KIR3DL2 and ILT2) and activated KIRs (KIR2DS4 and KIR3DS1) as open conformers (OCs) on NK cells (33–35). Also, receptors on NK cells are known to recognize peptides bound by HLA-F molecules or OC-type HLA-F molecules and function as immunoregulators (36). Moreover, a recent HCT study has indicated that soluble HLA-F might inhibit NK and T cells to favor allograft survival and that the upregulation of *HLA-F* might increase the severity of GVHD (37). Therefore, the HLA telomere genomic region that harbors *HLA-F* and *HLA-G* seems to be worth investigating in more detail to determine whether nucleotide sequence variants within the vicinity of the *HLA-F* and *HLA-G* gene loci are strongly associated with aGVHD.

In this study, to investigate whether novel MHC single-nucleotide polymorphisms (SNPs) might be associated with aGVHD, we performed the following: 1) analyzed a 460-kb haplotype block located within the telomeric region between *MAS1L* and *HLA-A* using SNP data of Japanese subjects in the International HapMap Project and the gene composition using gene mapping data from the National Center for Biotechnology Information (NCBI); 2) genotyped three loci, *OR2H2* (olfactory receptor family 2 subfamily H member 2), *HLA-F-AS1* (*HLA-F* antisense RNA 1), and *HLA-G*, that are located in the 460-kb haplotype block using 338 Japanese patient–donor pairs of unrelated bone marrow transplantation (UR-BMT) who were previously matched for *HLA-A*, *HLA-B*, *HLA-C*, *HLA-DRB1*, and *HLA-DQB1* (HLA-10/10); and 3) statistically analyzed the genotypes including *HLA-DPB1* with clinical and transplantation outcome by univariate and multivariate analyses.

## Materials and Methods

### Study Population

We selected 338 pairs (676 individuals) including patients with acute myeloid leukemia (AML) from 2,344 UR-BMT Japanese patient–donor pairs who previously were studied as part of the Japan Marrow Donor Program (JMDP) from 2006 to 2010 (17) for the present retrospective study using the following selection criteria: 1) patients transplanted from donors were *HLA-A*, *HLA-B*, *HLA-C*, *HLA-DRB1*, and *HLA-DQB1* matched at the field-2 level (HLA-10/10) and were *HLA-DPB1* matched or mismatched at the field-2 level in the GVH direction; 2) transplantation pairs were retyped for *HLA-A*, *HLA-B*, *HLA-C*, *HLA-DRB1*, *HLA-DQB1*, and *HLA-DPB1* alleles at the field-2 level PCR-SSOP or PCR-SBT methods (17); 3) transplanted non-T cell-depleted marrow were without *in vivo* use of anti-thymocyte globulin (ATG) for GVHD prophylaxis; 4) first transplantation; 5) Japanese ethnicity; and 6) survival was for more than 7 days after transplantation. The patient and donor characteristics of the 338 pairs are shown in **Table 1**; 193 were *HLA-DPB1* mismatched; 160 were diagnosed as grade 0, 81 as grade I, 67 as grade II, 26 as grade III, and 4 as grade IV.

**Table 1:** Patient and transplant characteristics.

Characteristics		(N = 338)
*HLA-DPB1* matching (match/mismatch)*		145/193
Median patient age, years (range)		49 (1–69)
Median donor age, years (range)		34 (20–56)
Gender matching (donor to patient), no. (%)		
Female to male		53 (15.7)
Male to male		140 (41.4)
Female to female		45 (13.3)
Male to female		100 (29.6)
Leukemia risk, no. (%)		
Standard		150 (44.4)
High		188 (55.6)
GVHD prophylaxis, no. (%)		
Cyclosporine based		78 (23.1)
Tacrolimus based		257 (76.0)
Others		3 (0.9)
Conditioning regimen, no. (%)		
Myeloablative		239 (70.7)
Reduced intensity		99 (29.3)

Leukemia risk: standard risk indicates the first or second complete remission (CR). Conditioning regimen: myeloablative regimen indicates conditioning regimens with total-body irradiation >8 Gy, oral busulfan ≥9 mg/kg, intravenous busulfan ≥7.2 mg/kg, or melphalan >140 mg/m^2^. Reduced intensity: reduced-intensity regimen indicates all other conditioning regimens.

N, number of patients; GVHD, graft-versus-host disease.

*GVH direction.

### Linkage Disequilibrium Analysis

**Figure 1:**
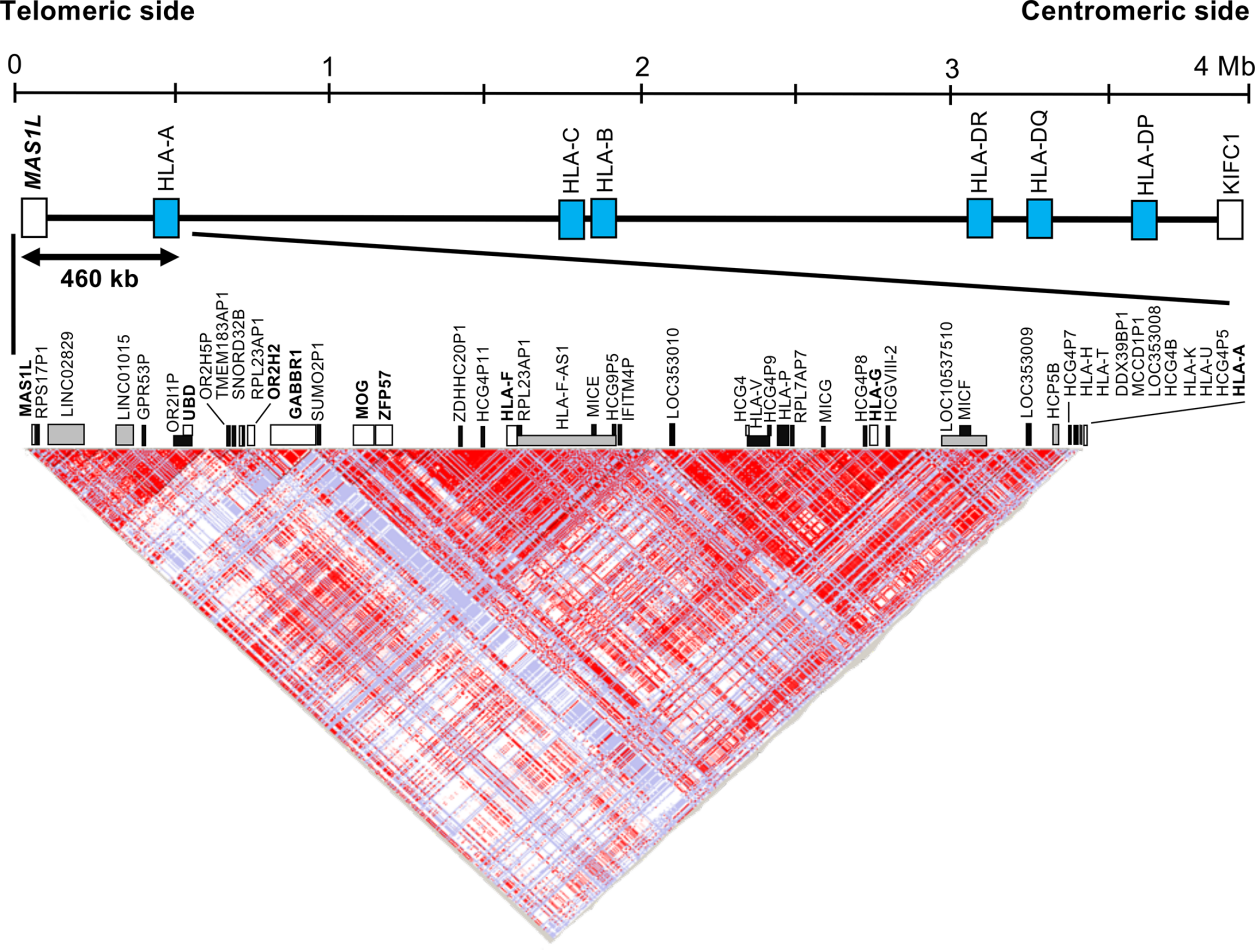
Haplotype structure of the human leukocyte antigen (HLA) telomeric genomic region between *MAS1L* and *HLA-A*. The linkage disequilibrium (LD) plot was generated with the program HaploView Ver.4.2. The top part shows the simplified gene composition of the 4-megabase (Mb) HLA region between *MAS1L* and *KIFC*. Blue box indicates representative classical HLA loci. The bottom part shows the LD map constructed by the HaploView software. A total of 603 single-nucleotide polymorphisms (SNPs) extracted from the HapMap data (hapmap3_r3) of 113 Japanese subjects in the 460-kb HLA telomeric genomic region between *MAS1L* and *HLA-A* (GRCh38.p13, chr6: 29486712 to 29946600) were used for this analysis. White, gray, and black boxes indicate coding genes, non-coding RNA (ncRNA), and pseudogenes, respectively. Each dot represents the value of D′/LOD, with the standard LD color scheme: white, LOD < 2 and D′ < 1; shade of pink/red, LOD > 2 and D′ < 1; blue, LOD < 2 and D′ = 1; bright red, LOD > 2 and D′ = 1.

Because an olfactory receptor gene cluster (ORGC) is located on the adjoining telomeric side of the *HLA-F* and *HLA-G* haplotype segment, we decided to include a portion of the ORGC genomic region from *MAS1L* to *HCG4P11* (**Figure 1**) as part of our linkage disequilibrium (LD) analysis of inferred polymorphic haplotypes. In this regard, we also chose to genotype the polymorphic olfactory receptor gene, *OR2H2*, within the ORGC genomic region as a potential, *a priori*, negative control. To understand the inferred haplotype structure in the 460-kb HLA telomeric genomic region (Genome Reference Consortium Human Build 38 patch release 13 (GRCh38.p13), chr6: 29486712 to 29946600) between *MAS1L* and *HLA-A*, we extracted all SNPs in this segment using the polymorphism data from 113 Japanese subjects analyzed by the International HapMap Project (hapmap3_r3, https://www.sanger.ac.uk/resources/downloads/human/hapmap3.html) (38). The LD map was constructed by HaploView software Ver.4.2 using the extracted SNPs and was used to narrow down genomic positions for designing DNA polymorphism markers (39).

### Search of Candidate Locations to Design Novel DNA Polymorphic Markers

Of the genomic DNA sequences that cover the entire HLA region derived from 95 HLA homozygous cell lines (40), we selected 43 haplotype sequences containing *HLA-A* alleles, which are frequently observed in the Japanese population (>0.2% allele frequency). After repeated sequences of the DNA sequences were masked by RepeatMasker software Ver. open-4.0.9 (http://www.repeatmasker.org), the unique DNA sequences between *MAS1L* and *HLA-A* were aligned by Sequencher Ver.5.0.1 DNA sequence assembly software (GENCODES, Ann Arbor, MI, USA). To perform effective genotyping by using a combination of PCR amplification and Sanger sequencing, we searched for genomic sites within the nucleotide alignments for a SNP density of more than five SNPs concentrated within less than 1 kb of sequence.

### Genotyping of OR2H2 and HLA-F-AS1

The *OR2H2* and *HLA-F-AS1* locus-specific primers are shown in **Table S1**. The primers were designed by Primer Express Ver.2.0 (Thermo Fisher Scientific, Waltham, MA, USA). The predicted amplicon sizes were 484 bp for *OR2H2* and 1,036 bp for *HLA-F-AS1*. The 20 μl PCR mixture included 50 ng of genomic DNA, 0.5 units of PrimeSTAR GXL DNA Polymerase (1.25 U/μl, TaKaRa Bio, Otsu, Shiga, Japan), 4 μl of 5× PrimeSTAR GXL Buffer (5 mM of Mg^2+^), 1.6 μl of each dNTP (2.5 mM), and 1.0 μl (4 pmol/μl) of each primer. The cycling parameters were as follows: an initial denaturation with 94°C for 2 min followed by 30 cycles of 98°C/10 s, 60°C/15 s, and 68°C/1 min. PCRs were performed with the thermal cycler GeneAmp PCR system 9700 (Thermo Fisher Scientific, Foster City, CA, USA). Purified PCR products were sequenced directly with Big Dye Terminator Kit Ver.1.1 and ABI3130xl genetic analyzer (Thermo Fisher Scientific, Carlsbad, CA, USA). The DNA sequencing chromatogram data were analyzed by the Sequencher Ver.5.0.1 DNA sequence assembly software.

### Genotyping of HLA-G

Since *HLA-G* is known to have low polymorphism over the entire gene region, the DNA sequences for full-length *HLA-G* alleles are difficult to determine by Sanger sequencing and short-read-based next-generation sequencing (NGS). Therefore, *in-phase* full-length DNA sequences were determined by the PacBio RSII sequencer (Pacific Biosciences, Menlo Park, CA, USA) using the long-ranged PCR products amplified by the previously designed *HLA-G*-specific primers (amplicon size, 5.6 kb) (41). We prepared pre-sequence samples according to the method for “preparing SMRTbell library with PacBio barcode universal primer for multiplexing amplicons” (https://www.pacb.com/wp-content/uploads/Procedure-Checklist-Preparing-SMRTbell-Libraries-using-PacBio-Barcoded-Universal-Primers-for-Multiplexing-Amplicons.pdf). The subsequent processes such as sequencing, clustering, phasing, consensus building, post-processing, and evaluation of the consensus sequences were performed according to our previous report (42). The *HLA-G* alleles were assigned by comparing the consensus sequences obtained by single-molecule real-time (SMRT) sequencing and the *HLA-G* allelic sequences released from the IPD-IMGT/HLA database (https://www.ebi.ac.uk/ipd/imgt/hla/) (43). The DNA sequencing chromatogram data were analyzed by the Sequencher ver.5.0.1 DNA sequence assembly software. Novel *HLA-G* allele candidates and *HLA-G* homozygote candidates were validated by Sanger sequencing using the ABI PRISM 3130xl Genetic Analyzer (Thermo Fisher Scientific, Foster City, CA, USA).

### Statistical Analysis of Genotypes and Clinical and Transplantation Outcomes

To evaluate associations of the mismatch information obtained from the four loci (*HLA-G*, *HLA-F-AS1*, *HLA-DPB1*, and *OR2H2*) and the impact on aGVHD, chronic GVHD (cGVHD), leukemia relapse, and transplant-related mortality, we performed the univariate and multivariable competing risk regression analyses using the Stata version 14 (StataCorp, Texas, USA) (44). The cumulative incidence of aGVHD was assessed by a method described previously (45). Confounders considered were patient age (linear), donor age (linear), gender (donor–recipient pair), risk of leukemia relapse (standard and high), GVHD prophylaxis (cyclosporine-based regimen, tacrolimus-based regimen, and other regimens without cyclosporine or tacrolimus), and conditioning regimen (reduced-intensity conditioning and myeloablative conditioning). Conditioning regimens were classified as myeloablative when total body irradiation was >8 Gy, oral busulfan was ≥9 mg/kg, intravenous busulfan was ≥7.2 mg/kg, or melphalan was >140 mg/m^2^, in accordance with Giralt et al. (46).

### Calculation of Heterozygosity

Expected and observed heterozygosity values were calculated by Excel function using the number of homozygotes for each allele. p-Values were calculated by the chi-square test using the expected and observed heterozygosity values.

## Results

### Development of DNA Polymorphic Markers in the Human Leukocyte Antigen Telomeric Region

**Figure 1** and **Table S2** show an LD map between two SNPs using the 603 SNPs, and 49 gene loci identified in the HLA telomeric region between *MAS1L* and *HLA-A*, respectively. In this region, a relatively large LD block was observed between *SUMO2P1* and *HLA-A*, which was subdivided into two LD blocks: *LOC353010*–*HLA-A* block and *SUMO2P1*–*IFITM4P* block. *HLA-G* and *HLA-F* were included in the two different LD blocks: *LOC353010*–*HLA-A* block and *SUMO2P1*–*IFITM4P* block. Moreover, *OR2H2* located on the telomeric side of *HLA-F* was included in another LD block independent of the other two blocks (**Figure 1**).

We identified two novel DNA polymorphic markers, *HLA-F-AS1* and *OR2H2*, by comparative analysis among 43 genomic DNA sequences derived from HLA homozygous cell lines (40). *HLA-F-AS1* is defined as a long non-coding RNA (lncRNA) located on the antisense side of *HLA-F* (Gene ID: 285830 in NCBI) (**Figure S1**), and seven known reference SNPs and seven haplotypes of *HLA-F-AS1* were identified in the 43 genomic DNA sequences (**Table 2**). The *OR2H2* SNP-dense site was composed of six known reference SNPs and six haplotypes (**Table 2**). Since *HLA-G* is included in the LD block adjoining *HLA-F-AS1*, we also selected this site as one of the three polymorphic DNA markers for genotyping UR-HCT cases with aGVHD. The physical distance between the three polymorphic markers is 140 kb between *OR2H2* and *HLA-F-AS1*, 80 kb between *HLA-F-AS1* and *HLA-G*, and 110 kb between *HLA-G* and *HLA-A*. A fourth polymorphic DNA marker, *HLA-DPB1*, in the HLA class II region (not shown) was included also for genotyping in this study.

**Table 2:** Characteristics of *HLA-F-AS1* and *OR2H2* DNA polymorphic markers.

*(A) HLA-F-AS1*								
*HLA-F-AS1* haplotype	Chr6 position of GRCh38.p13 (top row) and reference SNP ID (bottom row)							Number of haplotypes
	29742603	29742790	29742851	2974294	29742973	29743054	29743056	
	rs885941	rs115635977	rs730858	rs4713240	rs117584100	rs1610612	rs2076176	
HLA-F-AS1*01	C	C	G	G	A	G	C	15
HLA-F-AS1*02	C	C	A	A	A	G	C	10
HLA-F-AS1*03	T	C	G	A	A	A	G	9
HLA-F-AS1*04	C	G	G	A	A	G	G	3
HLA-F-AS1*05	C	C	G	A	A	G	G	3
HLA-F-AS1*06	T	C	G	A	A	G	G	2
HLA-F-AS1*07	T	C	G	A	G	A	G	1
**(B) *OR2H2***								
***OR2H2*** **haplotype**	**Chr6 position of GRCh38.p13 (top row) and SNP ID (bottom row)**							**Number of haplotypes**
	**29587796**	**29587808**	**29587904**	**29587961**	**29588001**	**29588032**		
	**rs17184360**	**rs17184367**	**rs3095275**	**rs61744969**	**rs2235698**	**rs3129034**		
OR2H2*01	C	A	G	C	A	T		16
OR2H2*02	C	A	G	C	A	C		13
OR2H2*03	C	G	G	C	G	T		9
OR2H2*04	C	A	A	C	A	C		2
OR2H2*05	T	A	A	C	A	C		2
OR2H2*06	C	G	G	A	G	T		1

GRCh38.p13: Genome Reference Consortium Human Build 38 patch release 13 from National Center for Biotechnology Information (NCBI; https://www.ncbi.nlm.nih.gov). Number of haplotypes: number detected in the 43 cell line sequences published by (40).

SNP, single-nucleotide polymorphism.

### Polymorphisms of OR2H2, HLA-F-AS1, HLA-G, and HLA-DPB1

From the polymorphism analyses of *OR2H2* and *HLA-F-AS1* markers using the 338 pairs (676 individuals), six and seven haplotypes were detected, respectively (GenBank Accession Nos. LC662707–LC662712 in *OR2H2* and LC662713–LC662719 in *HLA-F-AS1*, **Tables S3A, B**). These were the same as the *HLA-F-AS1* and *OR2H2* haplotypes detected for the 43 genomic DNA sequences derived from HLA homozygous cell lines (**Table 2**). There was no statistical bias in the haplotype frequencies between donor and patient for both *OR2H2* and *HLA-F-AS1*.

From the polymorphism analysis of *HLA-G* using the 338 pairs (676 individuals), 21 field-4 level (full-length level) allelic DNA sequences were identified (LC662720–LC662737 and LC662739–LC662741). By comparing these HLA-G allele sequences with those already within the IMGT/HLA database, we identified 12 novel alleles and 10 extended alleles (3,573–3,594 bp) that were in longer gene regions than in those that were previously determined (822–3,138 bp) (**Table S4**). The 14-bp indel polymorphism site (rs371194629) was included in all *HLA-G* allelic sequence analyses, and therefore the correspondence between the coding region’s polymorphisms and the 14-bp indel polymorphisms was clarified in all individuals. To investigate the association between *HLA-G* polymorphisms and aGVHD in detail, we created three datasets from the full-length level *HLA-G* sequences: the field-2 level alleles (*HLA-G_Field-2*), indel alleles in rs371194629 (*HLA-G_ Indels*), and haplotypes that combined both of them (*HLA-G_Field-2+Indels*). Seven, two, and eight alleles or haplotypes were detected in *HLA-G_Field-2*, *HLA-G_Indels*, and *HLA-G_Field-2+Indels*, respectively (**Tables S3C–E**). Furthermore, amino acid sequence polymorphisms deduced from the *HLA-G* alleles were not found in amino acid residues 100 and 103, which bind to KIR2DL4, and in residues 217–222 and 272, which bind to LILRB1/2 (47, 48).

In addition to *OR2H2*, *HLA-F-AS1*, and *HLA-G*, we performed an association analysis of *HLA-DPB1* that included mismatched cases. Fourteen *HLA-DPB1* alleles were detected from the previously genotyped *HLA-DPB1* of the 338 pairs (**Table S3F**).

Heterozygosity values of *OR2H2*, *HLA-F-AS1*, *HLA-G_Field-2*, *HLA-G_Indels*, *HLA-G_Field-2+Indels*, and *HLA-DPB1* were 0.73, 0.76, 0.49, 0.30, 0.57, and 0.85, respectively, and there was no statistically significant difference between the observed and expected values of heterozygotes (**Table 3**). Since these values suggest that allelic dropouts did not occur during PCR amplification, we proceeded with the following statistical analyses using genotype data of the four loci.

**Table 3:** Heterozygosity of *OR2H2*, *HLA-F-AS1*, *HLA-G*, and *HLA-DPB1*.

Locus name	Variable	Number of alleles or haplotypes	Heterozygosity		
			H*o*	H*e*	P-value
*OR2H2*	*OR2H2*	6	0.73	0.71	0.97
*HLA-F-AS1*	*HLA-F-AS1*	7	0.76	0.73	1.00
*HLA-G*	*HLA-G_Field-2*	7	0.49	0.49	1.00
*HLA-G*	*HLA-G_ Indels*	2	0.30	0.30	0.99
*HLA-G*	*HLA-G_Field-2+Indels*	8	0.57	0.57	1.00
*HLA-DPB1*	*HLA-DPB1*	14	0.85	0.79	1.00

Variable: for HLA-G locus, three datasets were created from full-length DNA sequences and used for this study. HLA-G_Field-2: the field-2 level (formerly known as 4-digit typing) alleles. HLA-G_Indels: 14 bp inserted or deleted alleles (rs371194629) in the 3′-untranslated region (3′UTR) of HLA-G. HLA-G_Field-2+Indels: haplotypes combining HLA-G_Field-2 and HLA-G_Indels. Ho: observed heterozygosity. He: expected heterozygosity.

### Association of OR2H2, HLA-F-AS1, HLA-G, and HLA-DPB1 Polymorphisms With Acute Graft-Versus-Host Disease

We initially evaluated mismatches between patient–donor pairs in the four loci of *OR2H2*, *HLA-F-AS1*, *HLA-G*, and *HLA-DPB1* and the effects of transplant prognosis by the univariate analysis. From the evaluation of the association between the mismatches in GVH direction and aGVHD, the *HLA-F-AS1* mismatch showed a high risk with grade III–IV and grade II–IV aGVHD (hazard ratio (HR), 2.89; 95% CI, 1.38–6.03; p = 0.005; and HR, 1.82; 95% CI, 1.16–2.85; p = 0.009, respectively). Similarly, the *HLA-G* (HLA-G_Field-2 and *HLA-G_Field-2+Indels*) and *HLA-DPB1* mismatches showed high risk of grade III–IV (HR, 3.84; 95% CI, 1.52–9.75; p = 0.005; and HR, 3.12; 95% CI, 1.35–7.20; p = 0.008) and grade II–IV (HR, 1.72; 95% CI, 1.12–2.64; p = 0.012), respectively (**Table 4**). In contrast, *OR2H2*, which is located at the most genomic, telomeric end of the polymorphic markers, and the *HLA-G_Indel* mismatches were not associated with aGVHD. Therefore, of the *HLA-G* dataset, the *HLA-G_Field-2*, which is related at a much stronger level to aGVHD than the *HLA-G_Field-2+Indels*, was used for the following multivariate analyses.

**Table 4:** Effect of *HLA-F-AS1*, *HLA-G*, and *HLA-DPB1* mismatches on aGVHD by the univariate analysis.

Variable		N	Grade III–IV						Grade II–IV				
			n	HR	[95% CI]		p-Value		n	HR	[95% CI]		p-Value
*OR2H2*	Match	253	19	1.00					67	1.00			
	Mismatch*	85	11	1.78	0.85	3.73	0.128		30	1.38	0.90	2.11	0.135
*HLA-F-AS1*	Match	279	19	1.00					72	1.00			
	Mismatch*	59	11	2.89	1.38	6.03	0.005		25	1.82	1.16	2.85	0.009
*HLA-G_Field-2*	Match	320	25	1.00					89	1.00			
	Mismatch*	18	5	3.84	1.52	9.75	0.005		8	1.76	0.87	3.56	0.116
*HLA-G_ Indels*	Match	324	28	1.00					91	1.00			
	Mismatch*	14	2	1.71	0.41	7.15	0.464		6	1.75	0.75	4.06	0.192
*HLA-G_Field-2+Indels*	Match	306	23	1.00					84	1.00			
	Mismatch*	32	7	3.12	1.35	7.20	0.008		13	1.63	0.91	2.92	0.100
*HLA-DPB1*	Match	145	9	1.00					31	1.00			
	Mismatch*	193	21	1.80	0.82	3.92	0.141		66	1.72	1.12	2.64	0.012

Variable: for HLA-G locus, three datasets were created from full-length DNA sequences and used for this study. HLA-G_Field-2: the field-2 level (formerly known as 4-digit typing) alleles. HLA-G_Indels: 14 bp inserted or deleted alleles (rs371194629) in the 3′-untranslated region (3′UTR) of HLA-G. HLA-G_Field-2+Indels: haplotypes combining HLA-G_Field-2 and HLA-G_Indels.

N, number of patients; n, number of grade III–IV or grade II–IV cases; HR, hazard ratio; aGVHD, acute graft-versus-host disease.

*GVH direction.

Multivariate analysis of *HLA-F-AS1*, *HLA-G*, and *HLA-DPB1* mismatches with clinical and transplantation results showed that the *HLA-F-AS1* and *HLA-DPB1* mismatches were strongly associated with grade II–IV aGVHD (HR, 1.76; 95% CI, 1.07–2.88; p = 0.026; and HR, 1.59; 95% CI, 1.02–2.49; p = 0.042) (**Table 5**). In contrast to the univariate analysis described above, the *HLA-G* mismatch did not show statistical significance in this multivariate analysis.

**Table 5:** Effect of *HLA-F-AS1*, *HLA-G*, and *HLA-DPB1* mismatches on transplantation outcome by the multivariate analysis.

Variable		N	Grade III–IV						Grade II–IV				
			n	HR	[95% CI]		p-Value		n	HR	[95% CI]		p-Value
*HLA-F-AS1*	Match	279	19	1.00					72	1.00			
	Mismatch*	59	11	2.11	0.83	5.33	0.115		25	1.76	1.07	2.88	0.026
*HLA-G_Field-2*	Match	320	25	1.00					89	1.00			
	Mismatch*	18	5	2.20	0.62	7.76	0.221		8	1.32	0.61	2.87	0.484
*HLA-DPB1*	Match	145	9	1.00					31	1.00			
	Mismatch*	193	21	1.63	0.73	3.65	0.234		66	1.59	1.02	2.49	0.042
Patient age (years)	1–68 (med. 49)	Linear	30	0.98	0.95	1.00	0.062		97	1.00	0.98	1.01	0.734
Donor age (years)	20–57 (med. 34)	Linear	30	1.03	0.98	1.08	0.242		97	1.02	0.99	1.05	0.196
Leukemia risk	Standard	150	9	1.00					42	1.00			
	High	188	21	1.64	0.71	3.79	0.246		55	0.91	0.60	1.37	0.645
GVHD prophylaxis	Cyclosporine based	78	8	1.00					27	1.00			
	Tacrolimus based	257	22	0.94	0.37	2.41	0.899		69	0.82	0.52	1.31	0.410
	Others	3	0	ND	ND	ND	ND		1	0.87	0.10	7.59	0.902
Conditioning regimen	Myeloablative	239	20	1.00					69	1.00			
	Reduced intensity	99	10	1.43	0.62	3.28	0.402		28	0.92	0.57	1.48	0.733
Gender matching (donor to patient)	Female to male	53	2	1.00					12	1.00			
	Male to male	140	15	2.86	0.69	11.85	0.148		40	1.26	0.64	2.45	0.505
	Female to female	45	3	1.91	0.33	11.09	0.472		11	1.20	0.52	2.78	0.673
	Male to female	100	10	2.12	0.47	9.54	0.329		34	1.51	0.77	2.96	0.227

HLA-G_Field-2: the field-2 level (formerly known as 4-digit typing) alleles;

N, number of patients; n, number of aGVHD grade III–IV or grade II–IV cases; med, median; HR, hazard ratio; ND, not detected; GVHD, graft-versus-host disease.

*GVH direction.

We performed a subgroup analysis of the risk by *HLA-F-AS1*, *HLA-G*, and/or *HLA-DPB1* on aGVHD, which is shown in **Table 6**. The *HLA-F-AS1* mismatch and *HLA-F-AS1/HLA-DPB1* dual mismatches showed significant effects on grade II–IV aGVHD and/or grade III–IV aGVHD. These results suggest that the *HLA-F-AS1* and *HLA-DPB1* mismatches may be involved not only in the development of grade II–IV aGVHD but also in the development of the more severe grade III–IV aGVHD. Although the possibility of an *HLA-G* mismatch having an effect on grade II–IV and III–IV aGVHD is not conclusive because of the small number of mismatch samples in our study, it was nevertheless statistically significant (p < 0.05) in three of four mismatched categories (**Table 6**). The high HR values ranging between 8.11 and 13.41 for *HLA-G* alone or associated with *HLA-F-AS1* and *HLA-DPB1* mismatches and grade III–IV aGVHD suggest a possible haplotypic role for *HLA-G* with adverse clinical impacts.

**Table 6:** Stratified analysis of *HLA-F-AS1*, *HLA-G*, and *HLA-DPB1* matching on aGVHD.

Variable			N	Grade III–IV						Grade II–IV				
*HLA-F-AS1*	*HLA-G_Field-2*	*HLA-DPB1*		n	HR	[95% CI]		p-Value		n	HR	[95% CI]		p-Value
Match	Match	Match	120	4	1.00					24	1.00			
Mismatch*	Match	Match	18	4	7.07	1.81	27.70	0.005		5	1.34	0.54	3.34	0.528
Match	Mismatch*	Match	4	1	8.11	0.97	67.70	0.053		1	1.21	0.18	8.16	0.842
Match	Match	Mismatch*	152	13	2.61	0.85	8.05	0.094		46	1.57	0.96	2.58	0.074
Mismatch*	Mismatch*	Match	3	0	ND	ND	ND	ND		1	1.86	0.24	14.46	0.555
Match	Mismatch*	Mismatch*	3	1	13.41	1.21	148.24	0.034		1	1.96	0.23	17.04	0.542
Mismatch*	Match	Mismatch*	30	4	4.31	1.06	17.62	0.042		14	2.87	1.46	5.63	0.002
Mismatch*	Mismatch*	Mismatch*	8	3	12.28	2.98	50.53	0.001		5	3.89	1.58	9.56	0.003

HLA-G_Field-2: the field-2 level (formerly known as 4-digit typing) alleles.

N, number of patients; n, number of aGVHD grade III–IV or grade II–IV cases; HR, hazard ratio; ND, not detected; aGVHD, acute graft-versus-host disease.

*GVH direction.

**Figure 2:**
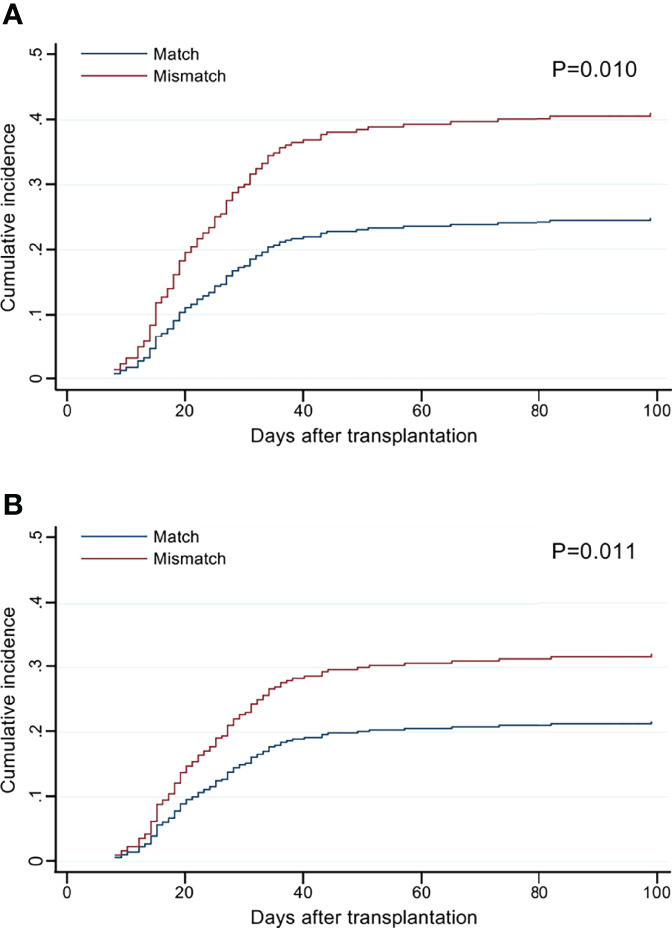
Cumulative incidence of grade II–IV acute graft-versus-host disease (aGVHD) by the mismatch number of *HLA-F-AS1* and *HLA-DPB1* at the allele level in the GVH direction. **(A, B)** Cumulative incidence curves of *HLA-F-AS1* and *HLA-DPB1*, respectively. Cumulative incidences at 100 days were *HLA-F-AS1* match, 26% (95% CI, 21%–31%); *HLA-F-AS1* mismatch, 42% (30%–55%); *HLA-DPB1* match, 21% (15%–28%); and *HLA-DPB1* mismatch, 34% (28%–41%).

The cumulative incidence curves of grade II–IV aGVHD by the number of *HLA-F-AS1* and *HLA-DPB1* locus mismatches showed clear risk differences compared to the matched cases (**Figure 2**).

### Association of OR2H2, HLA-F-AS1, HLA-G, and HLA-DPB1 Polymorphisms With Chronic Graft-Versus-Host Disease, Relapse, and Mortality

The multivariate analysis of *HLA-F-AS1*, *HLA-G*, and *HLA-DPB1* mismatches with cGVHD, relapse, and mortality showed that only *HLA-DPB1* tended to associate with cGVHD (HR, 1.58; 95% CI, 1.00–2.50; p = 0.050) but not with relapse and mortality (**Table S5**).

### Association Between the Genotypes of HLA-G_Indels and Acute Graft-Versus-Host Disease

Association analysis of *HLA-G_Indels* showed a risk of grade II–IV aGVHD in patients with the deletion/insertion (Del/Ins) genotype (HR, 1.63; 95% CI, 1.07–2.49; p = 0.024) (**Table 7**). In contrast, there were no significant differences between the insertion allele and aGVHD in donors.

**Table 7:** Association between the genotypes of *HLA-G_Indels* (rs371194629) and aGVHD.

Patient												
rs371194629Genotype	N	Grade III–IV						Grade II–IV				
		n	HR	[95% CI]		p-Value		n	HR	[95% CI]		p-Value
Del/Del	223	18	1.00					58	1.00			
Del/Ins	102	12	1.56	0.78	3.09	0.206		37	1.63	1.07	2.49	0.024
Ins/Ins	13	0	ND	ND	ND	ND		2	0.53	0.13	2.24	0.387
**Donor**												
**rs371194629 Genotype**	**N**	**Grade III–IV**						**Grade II–IV**				
		**n**	**HR**	**[95% CI]**		**p-Value**		**n**	**HR**	**[95% CI]**		**p-Value**
Del/Del	228	20	1.00					62	1.00			
Del/Ins	98	10	1.23	0.58	2.57	0.590		33	1.39	0.91	2.15	0.131
Ins/Ins	12	0	ND	ND	ND	ND		2	0.63	0.15	2.69	0.532

Del, deletion; Ins, insertion; HR, hazard ratio; ND, not detected; aGVHD, acute graft-versus-host disease.

## Discussion

In this case–control study of the HLA telomeric region using the 338 UR-BMT pairs, we discovered that the *HLA-F-AS1* mismatch showed a relatively strong association with aGVHD. The *HLA-F-AS1* mismatch showed a similar aGVHD risk (HR, 1.76 vs. 1.59) as *HLA-DPB1* (**Table 5**). However, no confounding was observed between the two polymorphic DNA markers (p = 0.512), which suggests that their effects on aGVHD are independent of different genetic factors (**Table S6**). Therefore, a novel aGVHD-related polymorphism appears to be located on the genomic segment of *HLA-F-AS1* or its neighborhood. Also, the mismatches of *HLA-F-AS1* and/or *HLA-DPB1* together may have an effect on the development of both grade II–IV and grade III–IV aGVHD.

We chose to genotype the polymorphic olfactory receptor gene, *OR2H2*, within the ORGC genomic region telomeric of *HLA-F-AS1* (**Figure 1**) as a potential, *a priori*, negative control. In this regard, the genotyped *OR2H2* marker located within the ORGC on the most telomeric side of *HLA-F-AS1* did not show an association with aGVHD, confirming that the significant SNP associations with aGVHD are located within a 330-kb region on the centromeric side of *OR2H2*.

In the HLA genomic region, duplication units, including HLA class I genes, *HLA-F-AS1*, and repetitive sequences such as retrotransposons, formed by segmental duplication events are densely distributed in the telomere side of *HLA-A* (22, 49). From our genomic sequencing of the primate MHC regions, we previously suggested that the MHC diversity is not limited to antigen/T-cell receptor (TCR) interacting sites of the classical HLA class I molecules but spreads to the surrounding genomic segments as “hitchhiking diversity” owing to the accumulated effect of overdominant selection acting on the classical HLA loci (41). Harmless hitchhiking mutations generated during primate evolution may have been changed pathologically in modern humans by structural and physiological changes and/or life–environmental changes. In fact, several disease-related genes, such as diffuse panbronchiolitis, psoriasis vulgaris, rheumatoid arthritis, and sarcoidosis, were identified in the hitchhiking areas (50). *HLA-F-AS1* is included in the hitchhiking area, along with *MICA* (51, 52), *HLA-G* (21), and the rs887464 SNP (53) associated with transplantation outcome. Also, some *HLA-F-AS1* variant(s) might be associated together as a haplotype with the *HLA-F* gene variant and *Alu-HF* and *SVA-HF* insertion variants that are located within close proximity (49). Therefore, uncharacterized aGVHD-related polymorphisms may still be present in the hitchhiking areas of the HLA genomic region.

Previously, SNP-based association studies in the HLA telomeric region were performed using the Affymetrix Human Mapping 500K array set and the 1,228 SNPs located in the HLA genomic region of the Illumina FastTrack Genotyping Service (Illumina, Inc.) (7, 53). However, the *HLA-F-AS1* polymorphism was not identified because the SNP set used for genotyping did not contain the seven SNPs composed of *HLA-F-AS1* markers. Association analyses of transplant recipients with aGVHD using microsatellite markers have also been reported, but the *HLA-F-AS1* genomic region has not been analyzed so far (54). Therefore, the identification of aGVHD-related polymorphisms may have been overlooked previously due to the extraordinarily complex genomic structure of the region.

Because *HLA-F-AS1* is defined as an lncRNA (55), its involvement with aGVHD appears to be different from the traditional notion of an antigen presentation system mediated by HLA molecules and their associated antigenic peptides. For example, *HLA-F-AS1* expression has been shown to influence the production and migration of macrophages *via* intermediates such as microRNA (miRNA) and profilin 1 in colorectal cancer (55, 56). Moreover, macrophage infiltration of skin lesions after HCT was suggested to be an important predictor of aGVHD as well as a negative prognostic factor of overall survival (OS) (57). Therefore, *HLA-F-AS1* may be associated with transplantation outcomes *via* macrophage production, migration, and infiltration and the regulation of HLA expression levels. However, since there is currently no direct evidence that *HLA-F-AS1* is a true aGVHD-related gene, detailed polymorphism analysis of the LD block containing the HLA-F-AS1 marker is still required to identify true aGVHD-related polymorphisms with stronger genetic effects. Also, validation of the association between *HLA-F-AS1* and aGVHD is necessary for another cohort because of the relatively small cohort (338 patient–donor pairs) that we used in this study. Furthermore, there may be ethnic differences that have a stronger effect on *HLA-F-AS1* than what was detected in the Japanese in the present study.

We also investigated the relationship between *HLA-G_Field-2* mismatch and aGVHD, and found a significant association of p = 0.005 for grade III–IV aGVHD but not grade II–IV by the univariate analysis (**Table 4**). This suggests a possible effect of the coding exons of *HLA-G* on severe aGVHD. However, there was no significant effect of an *HLA-G* allelic mismatch on the outcome of the transplantation (**Table 5**). However, the Del/Ins genotype of the 14-bp indels (rs371194629) in 3′UTR of the *HLA-G* was suggested to associate with the risk of grade II–IV aGVHD (**Table 7**). Here, we were unable to evaluate the effect of the Ins/Ins genotype on aGVHD because the number of Ins/Ins genotypes was inadequate in both the patient and donor groups. However, Boukouaci et al. (21) suggested that the *HLA-G* low expressor 14-bp insertion allele constitutes a risk factor for the incidence of severe aGVHD in patients who received bone marrow as a stem cell source (21). The rs371194629 is involved in the stability of *HLA-G* mRNAs (58), and the 14-bp indel polymorphism regulates the binding of multiple miRNAs such as *miR148* and *miR152*, along with neighborhood SNPs such as rs106332044 (59). Since these descriptions support our data, the insertion allele and Del/Ins genotype are considered a risk for aGVHD.

It seems that haplotype matching at all the gene loci of the MHC genomic region for unrelated HCT would be preferred to matching at only five HLA loci in order to reduce the risks of aGVHD (14, 15). MHC haplotype matching is not technically or financially feasible for most tissue and cell typing laboratories so at this time, matching at 5-HLA loci or haplo-identical (half-matched) remains the method of choice (60). Also, GVHD is still a significant and potentially life-threatening complication even when the HLA is matched at five or more loci for unrelated HCT (14, 15). To better understand the role of the *HLA-F-AS1*, *HLA-F*, and *HLA-G* independently or as a haplotypic segment in the development of aGVHD, we propose that future studies sequence this MHC genomic region by the PACBIO long-range sequencing method. A better understanding and definition of the haplotypes of the *HLA-F/HLA-G* genomic segment might be important in helping to reduce the risks of unrelated HCT and lead to more successful transplantation outcomes.

## Conclusion

This study revealed that *HLA-F-AS1* mismatches between sequence variants are significantly associated with the risk of developing grade II–IV aGVHD. The significant effect of the *HLA-F-AS1* mismatch on aGVHD was independent of the classical HLA and *HLA-G* polymorphisms. Thus, the results of this research provide useful new information for the development of future BMT donor selection algorithms and the coordination of patients and donors. We expect that our findings will lead to further new insights into a better understanding of the molecular mechanism of aGVHD caused by HLA-matched UR-BMT.

## Data Availability Statement

The datasets presented in this study can be found in online repositories. The names of the repository/repositories and accession number(s) can be found in the article/**Supplementary Material**.

## Ethics Statement

The studies involving human participants were reviewed and approved by Institutional Review Board for Clinical Research, Tokai University Ethics committee, JMDP. Written informed consent to participate in this study was provided by the participants’ legal guardian/next of kin.

## Author Contributions

SS, MT, UK, MM, YM, and TS participated in the design of the study. SS, AS, and SI performed nucleotide sequencing and genotyping. SS, SM, and YM performed statistical data analyses. SS, MT, JK, and TS performed the analysis and wrote the paper. All authors reviewed and approved the final version of the paper.

## Funding

This work was supported by the Japan Society for the Promotion of Science Grants-in-Aid for Scientific Research (KAKENHI) (16H06502 and 22K08465) and a Practical Research Project for Allergic Diseases and Immunology from the Japan Agency for Medical Research and Development (JP22ek0510032).

## Conflict of Interest

The authors declare that the research was conducted in the absence of any commercial or financial relationships that could be construed as a potential conflict of interest.

## Publisher’s Note

All claims expressed in this article are solely those of the authors and do not necessarily represent those of their affiliated organizations, or those of the publisher, the editors and the reviewers. Any product that may be evaluated in this article, or claim that may be made by its manufacturer, is not guaranteed or endorsed by the publisher.
