# Supplementary material for: Sequence Variations Within HLA-G and HLA-F Genomic Segments at the Human Leukocyte Antigen Telomeric End Associated With Acute Graft-Versus-Host Disease in Unrelated Bone Marrow Transplantation
Source: Front Immunol. 2022 Jul 21;13:938206. doi: 10.3389/fimmu.2022.938206 (PMC9351719; doi:10.3389/fimmu.2022.938206)
Supplement: Supplementary file 1 [file DataSheet_1.pdf]

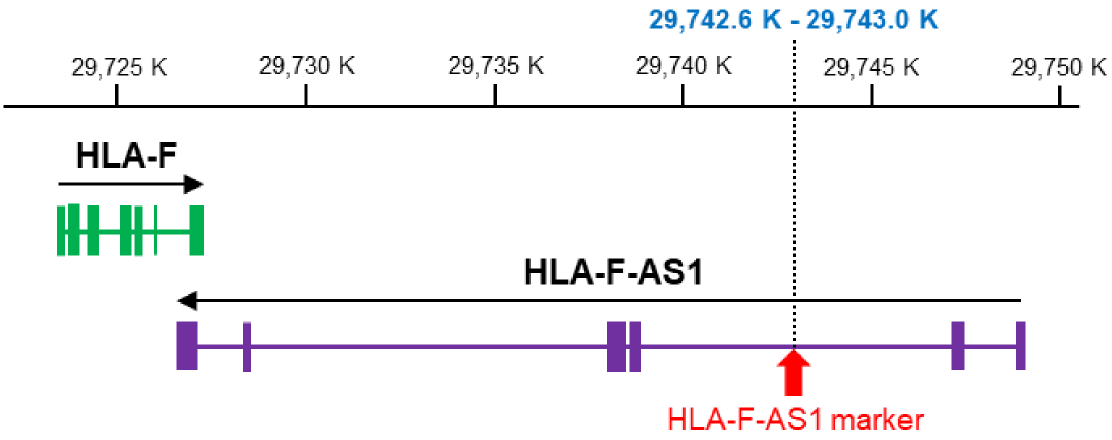

Supplementary Figure S1. Location of the *HLA-F-AS1* DNA polymorphic marker in the 25 kb of *HLA-F* and *HLA-AS1* genomic segment. Left side of the figure shows the telomere side of the short arm of chromosome 6. Position number is based on Genome Reference Consortium Human Build 38 patch release 13 (GRCh38.p13), and the exon and intron structures of *HLA-F* and *HLA-F-AS1* are based on the RNA sequences of NM_001098479.2 and NR_026972.1, respectively. Black and Red arrows indicate the gene direction and the location of *HLA-F-AS1* DNA polymorphic marker.
