# Supplementary material for: Sequence Variations Within HLA-G and HLA-F Genomic Segments at the Human Leukocyte Antigen Telomeric End Associated With Acute Graft-Versus-Host Disease in Unrelated Bone Marrow Transplantation
Source: Front Immunol. 2022 Jul 21;13:938206. doi: 10.3389/fimmu.2022.938206 (PMC9351719; doi:10.3389/fimmu.2022.938206)
Supplement: Supplementary file 2 [file DataSheet_2.pdf]

Supplementary Table S1. Primer list used for this study

Primer name	Primer sequence (5' to 3')	Application
OR2H2_PCR1	ACAGCATTCAGAACATAGTGGCA	for PCR
OR2H2_PCR2	AGGGTTAGGAGGTAGGAAGTG	for PCR
OR2H2_Seq	AGTGGCACTCAATAAATTTCCCC	for Sanger Sequencing
F-AS1_PCR1	CACCCAGGTCCATGACCTG	for PCR
F-AS1_PCR2	CGGTGCCCCTAGTCTCTTCC	for PCR
F-AS1_Seq	CCAAGTGTTACCTTGTCT	for Sanger Sequencing
HLA-G-Forward	**GCAGTCGAACATGTAGCTGACTCAGGTCAC** TTACATCCATCTACAGAGCCTCGC	for 1st PCR of SMRT sequencing
HLA-G-Reverse	**TGGATCACTTGTGCAAGCATCACATCGTAG** TAGCAGACAACAAATGCCACTTGA	for 1st PCR of SMRT sequencing

Bold letter indicates universal sequence required for the SMRT sequencing using the PacBio sequencer.
