# Supplementary material for: Sequence Variations Within HLA-G and HLA-F Genomic Segments at the Human Leukocyte Antigen Telomeric End Associated With Acute Graft-Versus-Host Disease in Unrelated Bone Marrow Transplantation
Source: Front Immunol. 2022 Jul 21;13:938206. doi: 10.3389/fimmu.2022.938206 (PMC9351719; doi:10.3389/fimmu.2022.938206)
Supplement: Supplementary file 3 [file DataSheet_3.pdf]

**Supplementary Table S2. Locus information in the HLA telomeric region between *MAS1L* to *HLA-A***

| Official Symbol | Official Full Name | GRCh38 chr6, position | Gene type | Strand |
|---|---|---|---|---|
| *MAS1L* | MAS1 proto-oncogene like, G protein-coupled receptor | 29486697-29487956 | protein coding | - |
| *RPS17P1* | Ribosomal protein S17 pseudogene 1 | 29489203-29489693 | pseudo | - |
| *LINC02829* | Long intergenic non-protein coding RNA 2829 | 29497475-29510558 | ncRNA | + |
| *LINC01015* | Long intergenic non-protein coding RNA 1015 | 29529406-29533568 | ncRNA | + |
| *GPR53P* | G protein-coupled receptor 53, pseudogene | 29537812-29538651 | pseudo | - |
| *OR2I1P* | Olfactory receptor family 2 subfamily I member 1 pseudogene | 29553105-29554163 | pseudo | + |
| *UBD* | Ubiquitin D | 29555515-29559732 | protein coding | + |
| *OR2H5P* | Olfactory receptor family 2 subfamily H member 5 pseudogene | 29574073-29574651 | pseudo | + |
| *TMEM183AP1* | Transmembrane protein 183A pseudogene 1 | 29577459-29577748 | pseudo | + |
| *SNORD32B* | Small nucleolar RNA, C/D box 32B | 29582252-29582328 | snoRNA | + |
| *RPL13AP* | Ribosomal protein L13a pseudogene | 29582508-29583055 | pseudo | + |
| *OR2H2* | Olfactory receptor family 2 subfamily H member 2 | 29585121-29590506 | protein coding | + |
| *GABBR1* | Gamma-aminobutyric acid type B receptor subunit 1 | 29602230-29633183 | protein coding | - |
| *SUMO2P1* | SUMO2 pseudogene 1 | 29635454-29636468 | pseudo | - |
| *MOG* | Myelin oligodendrocyte glycoprotein | 29657092-29672365 | protein coding | + |
| *ZFP57* | ZFP57 zinc finger protein | 29672392-29681152 | protein coding | - |
| *ZDHHC20P1* | Zinc finger DHHC-type containing 20 pseudogene 1 | 29708141-29708547 | pseudo | - |
| *HCG4P11* | HLA complex group 4 pseudogene 11 | 29721178-29722167 | pseudo | - |
| *HLA-F* | Major histocompatibility complex, class I, F | 29723434-29740355 | protein coding | + |
| *RPL23AP1* | Ribosomal protein L23a pseudogene 1 | 29726597-29727154 | pseudo | - |
| *HLA-F-AS1* | HLA-F antisense RNA 1 | 29726601-29749049 | ncRNA | - |
| *MICE* | MHC class I polypeptide-related sequence E | 29741556-29745784 | pseudo | - |
| *HCG9P5* | HLA complex group 9 pseudogene 5 | 29748254-29748648 | pseudo | + |
| *IFITM4P* | Interferon induced transmembrane protein 4 pseudogene | 29750807-29751148 | pseudo | - |
| *LOC353010* | HLA complex group 26 (non-protein coding) pseudogene | 29765116-29766296 | pseudo | - |
| *HCG4* | HLA complex group 4 | 29791031-29793073 | ncRNA | - |
| *HLA-V* | Major histocompatibility complex, class I, V | 29791906-29797807 | pseudo | + |

| | | | | |
|---|---|---|---|---|
| *HCG4P9* | HLA complex group 4 pseudogene 9 | 29798424-29800126 | pseudo | - |
| *HLA-P* | Major histocompatibility complex, class I, P | 29800044-29803079 | pseudo | + |
| *RPL7AP7* | Ribosomal protein L7a pseudogene 7 | 29803132-29804017 | pseudo | - |
| *MICG* | MHC class I polypeptide-related sequence G | 29812390-29812692 | pseudo | - |
| *HCG4P8* | HLA complex group 4 pseudogene 8 | 29826129-29827114 | pseudo | - |
| *HLA-G* | Major histocompatibility complex, class I, G | 29826474-29831130 | protein coding | + |
| *HCGVIII-2* | HCGVIII-2 pseudogene | 29833588-29835128 | pseudo | - |
| *LOC10537510* | uncharacterized LOC105375010 | 29848002-29857386 | ncRNA | - |
| *MICF* | MHC class I polypeptide-related sequence F | 29852187-29854052 | pseudo | - |
| *LOC353009* | HLA complex group 26 (non-protein coding) pseudogene | 29865915-29867087 | pseudo | - |
| *HCP5B* | HLA complex P5B | 29871895-29873783 | ncRNA | - |
| *HCG4P7* | HLA complex group 4 pseudogene 7 | 29886110-29887007 | pseudo | - |
| *HLA-H* | Major histocompatibility complex, class I, H | 29887573-29891079 | pseudo | + |
| *HLA-T* | Major histocompatibility complex, class I, T | 29896443-29898947 | pseudo | + |
| *DDX39BP1* | DEAD-box helicase 39B pseudogene 1 | 29906547-29907449 | pseudo | + |
| *MCCD1P1* | Mitochondrial coiled-coil domain 1 pseudogene 1 | 29907783-29908645 | pseudo | - |
| *LOC353008* | HLA complex group 26 (non-protein coding) pseudogene | 29910235-29911400 | pseudo | - |
| *HCG4B* | HLA complex group 4B | 29924592-29927215 | ncRNA | - |
| *HLA-K* | Major histocompatibility complex, class I, K | 29926659-29929825 | pseudo | + |
| *HLA-U* | Major histocompatibility complex, class I, U | 29933764-29934880 | pseudo | + |
| *HCG4P5* | HLA complex group 4 pseudogene 5 | 29940912-29941801 | pseudo | - |
| *HLA-A* | Major histocompatibility complex, class I, A | 29942532-29945870 | protein coding | + |

GRCh38.p13: Genome Reference Consortium Human Build 38 patch release 13 from National Center for Biotechnology Information (NCBI; https://www.ncbi.nlm.nih.gov); white background: protein coding gene; gray background: non-coding RNA (ncRNA) or small nucleolar RNA (snoRNA); and black background: pseudogene.
