# Supplementary material for: Sequence Variations Within HLA-G and HLA-F Genomic Segments at the Human Leukocyte Antigen Telomeric End Associated With Acute Graft-Versus-Host Disease in Unrelated Bone Marrow Transplantation
Source: Front Immunol. 2022 Jul 21;13:938206. doi: 10.3389/fimmu.2022.938206 (PMC9351719; doi:10.3389/fimmu.2022.938206)
Supplement: Supplementary file 4 [file DataSheet_4.pdf]

**Supplementary Table S3. Allele or haplotype frequency of *OR2H2, HLA-F-AS1, HLA-G* and *HLA-DPB1***

**(A) *OR2H2***

| Haplotype | N | | | Frequency of total number | P-value |
|---|---|---|---|---|---|
| | Donor | Patient | Total | | |
| OR2H2*01 | 296 | 281 | 577 | 42.7% | 0.441 |
| OR2H2*02 | 18 | 21 | 39 | 2.9% | 0.746 |
| OR2H2*03 | 164 | 168 | 332 | 24.6% | 0.850 |
| OR2H2*04 | 58 | 62 | 120 | 8.9% | 0.774 |
| OR2H2*05 | 139 | 144 | 283 | 20.9% | 0.789 |
| OR2H2*06 | 1 | 0 | 1 | 0.1% | 1 |
| Total | 676 | 676 | 1352 | 100% | |

**(B) *HLA-F-AS1***

| Haplotype | N | | | Frequency of total number | P-value |
|---|---|---|---|---|---|
| | Donor | Patient | Total | | |
| HLA-F-AS1*01 | 158 | 142 | 300 | 22.2% | 0.326 |
| HLA-F-AS1*02 | 56 | 56 | 112 | 8.3% | 1 |
| HLA-F-AS1*03 | 215 | 223 | 438 | 32.4% | 0.684 |
| HLA-F-AS1*04 | 2 | 4 | 6 | 0.4% | 0.687 |
| HLA-F-AS1*05 | 216 | 219 | 435 | 32.2% | 0.907 |
| HLA-F-AS1*06 | 28 | 31 | 59 | 4.4% | 0.790 |
| HLA-F-AS1*07 | 1 | 1 | 2 | 0.1% | 1 |
| Total | 676 | 676 | 1352 | 100% | |

**(C) *HLA-G_Field-2***

| Allele | N | | | Frequency of total number | P-value |
|---|---|---|---|---|---|
| | Donor | Patient | Total | | |
| G*01:01 | 271 | 266 | 537 | 39.7% | 0.824 |
| G*01:03 | 3 | 4 | 7 | 0.5% | 1 |
| G*01:04 | 397 | 400 | 797 | 58.9% | 0.912 |
| G*01:04_S336G | 0 | 1 | 1 | 0.1% | 1 |
| G*01:05N | 1 | 1 | 2 | 0.1% | 1 |
| G*01:06 | 3 | 3 | 6 | 0.4% | 1 |
| G*01:21N | 1 | 1 | 2 | 0.1% | 1 |
| Total | 676 | 676 | 1352 | 100% | |

**(D) *HLA-G_Indels***

| Allele | N | | | Frequency of total number | P-value |
|---|---|---|---|---|---|
| | Donor | Patient | Total | | |
| Del | 554 | 548 | 1102 | 81.5% | 0.726 |
| Ins | 122 | 128 | 250 | 18.5% | 0.726 |
| Total | 676 | 676 | 1352 | 100% | |

**(E)** ***HLA-G_Field-2+Indels***

| Haplotype | N | | | Frequency of total number | P-value |
|---|---|---|---|---|---|
| | Donor | Patient | Total | | |
| G*01:01+Del | 156 | 146 | 302 | 22.3% | 0.557 |
| G*01:04+Del | 397 | 400 | 797 | 58.9% | 0.912 |
| G*01:04_S336G+Del | 0 | 1 | 1 | 0.1% | 1 |
| G*01:21N+Del | 1 | 1 | 2 | 0.1% | 1 |
| G*01:01+Ins | 115 | 120 | 235 | 17.4% | 0.774 |
| G*01:03+Ins | 3 | 4 | 7 | 0.5% | 1 |
| G*01:05N+Ins | 1 | 1 | 2 | 0.1% | 1 |
| G*01:06+Ins | 3 | 3 | 6 | 0.4% | 1 |

**(F)** ***HLA-DPB1***

| Allele | N | | | Frequency of total number | P-value |
|---|---|---|---|---|---|
| | Donor | Patient | Total | | |
| DPB1*02:01 | 123 | 129 | 252 | 18.6% | 0.727 |
| DPB1*02:02 | 19 | 23 | 42 | 3.1% | 0.639 |
| DPB1*03:01 | 14 | 17 | 31 | 2.3% | 0.717 |
| DPB1*04:01 | 47 | 47 | 94 | 7.0% | 1 |
| DPB1*04:02 | 106 | 102 | 208 | 15.4% | 0.821 |
| DPB1*05:01 | 222 | 222 | 444 | 32.8% | 1 |
| DPB1*06:01 | 1 | 3 | 4 | 0.3% | 0.624 |
| DPB1*09:01 | 131 | 122 | 253 | 18.7% | 0.577 |
| DPB1*13:01 | 5 | 5 | 10 | 0.7% | 1 |
| DPB1*14:01 | 3 | 5 | 8 | 0.6% | 0.726 |
| DPB1*17:01 | 1 | 1 | 2 | 0.1% | 1 |
| DPB1*19:01 | 2 | 0 | 2 | 0.1% | 0.5 |
| DPB1*38:01 | 1 | 0 | 1 | 0.1% | 1 |
| DPB1*41:01 | 1 | 0 | 1 | 0.1% | 1 |
| Total | 676 | 676 | 1352 | 100% | |

N: Number of alleles; and P-value: Calculation by Fisher's exact test using the frequencies between patients and donor.
