# Supplementary material for: Sequence Variations Within HLA-G and HLA-F Genomic Segments at the Human Leukocyte Antigen Telomeric End Associated With Acute Graft-Versus-Host Disease in Unrelated Bone Marrow Transplantation
Source: Front Immunol. 2022 Jul 21;13:938206. doi: 10.3389/fimmu.2022.938206 (PMC9351719; doi:10.3389/fimmu.2022.938206)
Supplement: Supplementary file 5 [file DataSheet_5.pdf]

Supplementary Table S4. Characteristics of *HLA-G* polymorphisms

HLA-G allele name		14-bp indels (rs371194629)	GenBank accession No	Nucleotide length (bp)	Novel or Extended	Location of variation	Detected allele number		
Field-4 level	Field-2 level						Donor	Patient	Total
G*01:01:01:01	G*01:01	Deletion	LC662720	3,580	Extended		139	136	275
G*01:01:01:01-nov1	G*01:01	Deletion	LC662721	3,579	Novel	Intronic region	2	0	2
G*01:01:01:01-nov2	G*01:01	Deletion	LC662722	3,580	Novel	Intronic region	0	1	1
G*01:01:01:01-nov3	G*01:01	Deletion	LC662723	3,580	Novel	Intronic region	1	0	1
G*01:04:01:01-nov1	G*01:01*	Deletion	LC662727	3,580	Novel	Coding region	14	9	23
G*01:04:01:01	G*01:04	Deletion	LC662730	3,580	Extended		245	252	497
G*01:04:01:02	G*01:04	Deletion	LC662736	3,580	Extended		121	116	237
G*01:04:01:01-nov2	G*01:04	Deletion	LC662731	3,579	Novel	Intronic region	30	29	59
G*01:04:01:01-nov3	G*01:04	Deletion	LC662733	3,580	Novel	3'UTR	1	0	1
G*01:04:01:01-nov4	G*01:04	Deletion	LC662734	3,580	Novel	Intronic region	0	1	1
G*01:04:01:01-nov5	G*01:04	Deletion	LC662735	3,580	Novel	Intronic region	0	1	1
G*01:04:01:01-nov6	G*01:04	Deletion	LC662732	3,573	Novel/Extended	3'UTR	0	1	1
G*01:04nov_S336G	G*01:04nov_S336G	Deletion	LC662737	3,580	Novel	Coding region	0	1	1
G*01:21N	G*01:21N	Deletion	LC662741	3,580	Extended		1	1	2
G*01:01:02:01	G*01:01	Insertion	LC662724	3,594	Extended		82	85	167
G*01:01:03:03	G*01:01	Insertion	LC662726	3,594	Extended		30	33	63
G*01:01:02:01-nov	G*01:01	Insertion	LC662725	3,594	Novel	Intronic region	3	1	4
G*01:01:01:01-nov4	G*01:01	Insertion	LC662728	3,594	Novel	Coding region	0	1	1
G*01:03:01:02	G*01:03	Insertion	LC662729	3,594	Extended		3	4	7
G*01:05N	G*01:05N	Insertion	LC662739	3,593	Extended		1	1	2
G*01:06:01:01	G*01:06	Insertion	LC662740	3,594	Extended		3	3	6

*: The amino acid sequence of G*01nov_I134L is 100% identical to G*01:01.
