# Supplementary material for: Sequence Variations Within HLA-G and HLA-F Genomic Segments at the Human Leukocyte Antigen Telomeric End Associated With Acute Graft-Versus-Host Disease in Unrelated Bone Marrow Transplantation
Source: Front Immunol. 2022 Jul 21;13:938206. doi: 10.3389/fimmu.2022.938206 (PMC9351719; doi:10.3389/fimmu.2022.938206)
Supplement: Supplementary file 6 [file DataSheet_6.pdf]

Supplementary Table S5. Effect of mismatches on each DNA polymorphic markers and cGVHD, leukemia relapse and mortality by the univariate and multivariate analyses

(A) Univariate analysis

Variable		cGVHD**					N	Leukemia relapse				Mortality			
		N	HR	[95% C.I.]		P-value		HR	[95% C.I.]		P-value	HR	[95% C.I.]		P-value
OR2H2	Match	226	1.00				253	1.00				1.00			
	Mismatch*	78	0.99	0.60	1.63	0.969	85	1.31	0.83	2.07	0.242	0.93	0.62	1.39	0.724
HLA-F-AS1	Match	252	1.00				279	1.00				1.00			
	Mismatch*	52	0.63	0.33	1.21	0.161	59	1.03	0.59	1.80	0.905	0.95	0.59	1.53	0.836
HLA-G_Field-2	Match	287	1.00				320	1.00				1.00			
	Mismatch*	17	0.18	0.02	1.41	0.103	18	0.61	0.19	1.94	0.401	1.12	0.52	2.41	0.764
HLA-DPB1	Match	134	1.00				145	1.00				1.00			
	Mismatch*	170	1.48	0.94	2.32	0.088	193	0.99	0.65	1.51	0.953	1.09	0.76	1.54	0.648

(B) Multivariate analysis at three loci

Variable		cGVHD**					N	Relapse				Mortality			
		N	HR	[95% C.I.]		P-value		HR	[95% C.I.]		P-value	HR	[95% C.I.]		P-value
HLA-F-AS1	match	252	1.00				279	1.00				1.00			
	mismatch*	52	0.70	0.36	1.35	0.282	59	1.12	0.63	1.99	0.697	0.92	0.56	1.52	0.751
HLA-G_Field-2	match	287	1.00				320	1.00				1.00			
	mismatch*	17	0.20	0.03	1.51	0.119	18	0.58	0.17	1.90	0.365	1.16	0.52	2.57	0.713
HLA-DPB1	match	134	1.00				145	1.00				1.00			
	mismatch*	170	1.56	0.99	2.46	0.056	193	0.98	0.64	1.50	0.936	1.09	0.77	1.54	0.641

(C) Multivariate analysis at three loci with clinical information and transplantation outcome

Variable		cGVHD**					N	Relapse				Mortality			
		N	HR	[95% C.I.]		P-value		HR	[95% C.I.]		P-value	HR	[95% C.I.]		P-value
HLA-F-AS1	match	252	1.00				279	1.00				1.00			
	mismatch*	52	0.62	0.31	1.20	0.156	59	1.10	0.60	2.00	0.768	0.91	0.55	1.51	0.717

HLA-G_Field-2	match	287	1.00				320	1.00				1.00			
	mismatch*	17	0.25	0.03	1.94	0.187	18	0.42	0.13	1.41	0.161	0.92	0.41	2.06	0.834
HLA-DPB1	match	134	1.00				145	1.00				1.00			
	mismatch*	170	1.58	1.00	2.50	0.050	193	0.91	0.59	1.42	0.683	1.02	0.71	1.45	0.929
Patient age (y.o.)	1 - 68 (med. 49)	linear	1.00	0.98	1.02	0.960	linear	1.00	0.98	1.02	0.789	1.01	0.99	1.02	0.308
Donor age (y.o.)	20 - 57 (med. 34)	linear	0.99	0.96	1.02	0.380	linear	0.99	0.96	1.02	0.556	1.00	0.98	1.03	0.703
Leukemia risk	Standard	140	1.00				150	1.00				1.00			
	High	164	0.73	0.46	1.15	0.175	188	2.30	1.45	3.66	0.000	2.56	1.72	3.82	0.000
GVHD prophylaxis	Cyclosporin based	73	1.00				78	1.00				1.00			
	Tacrolimus based	229	0.91	0.55	1.51	0.711	257	0.91	0.55	1.53	0.733	1.14	0.75	1.74	0.528
	others	2	0.00	0.00	0.00	0.000	3	1.04	0.09	11.39	0.975	0.93	0.12	6.99	0.942
Conditioning regimen	myeloablative	218	1.00				239	1.00				1.00			
	reduced intensity	86	1.16	0.64	2.10	0.623	99	1.48	0.84	2.63	0.177	1.20	0.76	1.90	0.439
Gender matching (Donor to Patient)	Female to male	49	1.00				53	1.00				1.00			
	Male to male	122	1.89	0.88	4.04	0.103	140	0.73	0.40	1.35	0.320	0.82	0.50	1.34	0.424
	Female to female	40	1.74	0.73	4.12	0.209	45	1.05	0.49	2.26	0.894	0.96	0.51	1.78	0.888
	Male to female	93	1.57	0.72	3.45	0.258	100	0.83	0.44	1.58	0.566	0.72	0.42	1.24	0.239

*: GVH direction; **: Chronic GVHD (cGVHD) at 2 years was assessed in patients who survived 100 or more days after transplantation; HLA-G_Field-2: the field-2 level (formerly known as 4-digit typing) alleles; N: Number of patients; n: Number of Grade III-IV or Grade II-IV cases; y.o: Years old; med: Median; HR: Hazard ratio; and [95% C.I.]: 95% confidence interval.
