# Supplementary material for: Sequence Variations Within HLA-G and HLA-F Genomic Segments at the Human Leukocyte Antigen Telomeric End Associated With Acute Graft-Versus-Host Disease in Unrelated Bone Marrow Transplantation
Source: Front Immunol. 2022 Jul 21;13:938206. doi: 10.3389/fimmu.2022.938206 (PMC9351719; doi:10.3389/fimmu.2022.938206)
Supplement: Supplementary file 7 [file DataSheet_7.pdf]

Supplementary Table S6. Interaction analysis between two polymorphic markers in grade II-IV aGVHD

Interaction	HR	[95% C.I.]		P-value
HLA-F-AS1 vs *HLA-G_Field-2*	1.31	0.25	6.89	0.752
HLA-F-AS1 vs *HLA-DPB1*	1.40	0.51	3.81	0.512
HLA-G_Field-2 vs *HLA-DPB1*	1.17	0.23	5.85	0.852
HLA-F-AS1 vs *OR2H2*	1.05	0.37	2.93	0.930

HLA-G_Field-2: the field-2 level (formerly known as 4-digit typing) alleles; HR: Hazard ratio; and [95% C.I.]: 95% confidence interval.
